# Phylogenetically Diverse *Escherichia coli* Strains from Chicken Coharbor Multiple Carbapenemase-Encoding Genes (*bla*_NDM_*-bla*_OXA_-*bla*I_MP_)

**DOI:** 10.1155/2021/5596502

**Published:** 2021-10-06

**Authors:** Erkihun Aklilu, Azian Harun, Kirnpal Kaur Banga Singh, Shamsaldeen Ibrahim, Nor Fadhilah Kamaruzzaman

**Affiliations:** ^1^Faculty of Veterinary Medicine Universiti Malaysia Kelantan, Locked Box 36, Pengkalan Chepa 16100 Kota Bharu, Kelantan, Malaysia; ^2^Department of Medical Microbiology and Parasitology, School of Medical Sciences, Universiti Sains Malaysia, 16150 Kubang Kerian, Kota Bharu, Kelantan, Malaysia

## Abstract

Carbapenem-resistant Enterobacteriaceae (CRE) has been a public health risk in several countries, and recent reports indicate the emergence of CRE in food animals. This study was conducted to investigate the occurrence, resistance patterns, and phylogenetic diversity of carbapenem-resistant *E. coli* (CREC) from chicken. Routine bacteriology, PCR detection of *E. coli* species, multiplex PCR to detect carbapenemase-encoding genes, and phylogeny of CRE *E. coli* were conducted. The results show that 24.36% (19/78) were identified as CREC based on the phenotypic identifications of which 17 were positive for the tested carbapenemases genes. The majority, 57.99% (11/19), of the isolates harbored multiple carbapenemase genes. Four isolates harbored all *bla*_NDM_, *bla*_OXA_, and *bla*_IMP_, and five and two different isolates harbored *bla*_NDM_ and *bla*_OXA_ and *bla*_OXA_ and *bla*_IMP_, respectively. The meropenem, imipenem, and ertapenem MIC values for the isolates ranged from 2 *μ*g/mL to ≥256 *μ*g/mL. Phylogenetic grouping showed that the CREC isolates belonged to five different groups: groups A, B1, C, D, and unknown. The detection of CREC in this study shows that it has become an emerging problem in farm animals, particularly, in poultry farms. This also implies the potential public health risks posed by CRE from chicken to the consumers.

## 1. Introduction

Carbapenem resistance in Enterobacteriaceae is a serious emerging antimicrobial resistance (AMR) issue that has been escalating and posing challenges in treating infections caused by the resistant pathogen. Enterobacteriaceae are inhabitants of the intestinal flora and are among the most common human pathogens that cause cystitis and pyelonephritis with fever, septicemia, pneumonia, peritonitis, meningitis, and device-associated infections [1]. The bacteria are transmitted easily between human and animals, especially via fomites, food, and water. During the transmission, genetic materials are transferred through horizontal gene transfer, mediated mostly by plasmids and transposons. Enterobacteriaceae are among the common nosocomial pathogens often causing infections through medical devices that include ventilators, intravenous catheters, urinary catheters, or wounds caused by injury or surgery [2]. Such nosocomial infections commonly affect immunocompromised patients and in patients being treated using invasive devices.

Carbapenem is a broad-spectrum *β*-lactam antibiotic that is regarded as the last-line antibiotic, especially to be used in critically ill patients who have developed antimicrobial-resistant bacterial infections. Unfortunately, Enterobacteriaceae have developed resistance against this last resort drug and made it ever challenging to treat infections caused by these pathogens. Among the bacteria in the family Enterobacteriaceae, *E. coli*, and *Klebsiella pneumoniae* are the most commonly detected CRE that have been posing threat to the public health and animal health [3]. Such prevailing AMR issue has been compromising the efficacy of antibiotics, and according to the World Health Organization, there is a possibility for the world to encounter an era, in which all the antibiotics become ineffective thereby increasing mortality rate and increasing cost of treatment if no intervention is done to overcome the problem. There are also concerns that failure to counter the rising AMR problems worldwide may lead to reemergence of previously eradicated or controlled diseases [4].

According to the National Surveillance of Antimicrobial Resistance (NSAR) in Malaysia, from 2006 to 2017, which analyzed the data obtained from hospital microbiology laboratories from different parts of the country, carbapenem resistance in *E. coli* declined from 0.5% in 2010 to 0.2% in 2014 [5]. A recent report on the prevalence of CRE in a tertiary hospital in Malaysia shows that the prevalence of CRE in 2015 and 2016 was 0.3% (5/1590) and 1.2% (17/1402), respectively. The same study reported that the majority (81.8%) of the isolates were *Klebsiella pneumoniae*, followed by *Serratia marcescens*, *Escherichia coli*, and *Citrobacter koseri* [6]. A more recent study by Ghazali et al. [7] reported CRCE prevalence of 1% (2/200) from broiler chicken cloacal swab samples collected antemortem from abattoir in Terengganu. According to Zaidah et al. [8], unpublished data from different investigations in Malaysia indicated that the number of CRE isolated in general and tertiary hospitals is on the rising trend and is alarming. However, the data on the prevalence and molecular characteristics of CREC in food animals, particularly in chicken, are still scarce and not fully investigated. Therefore, this study was conducted to detect the presence of carbapenem-resistant *E. coli* in live chicken, investigate the antimicrobial resistance patterns, determine the phylogeny, and identify the common carbapenemase genes in the *E. coli* isolates.

## 2. Results

### 2.1. Bacterial Isolation and Identification and Antimicrobial Resistance Patterns

Based on the routine microbiology, 56.7% (85/150) of the cloacal swab samples were positive for *E. coli*. However, further confirmation using *E. coli* species-specific PCR showed that 78 out of the 85 (91.7%) isolates were identified as *E. coli*. Overall, the PCR results showed 52% (78/150) detection rate of *E. coli* from the cloacal swab samples collected. The resistance pattern of *E. coli* isolates showed that 87.18% were resistant to streptomycin, followed by ceftriaxone (80%), trimethoprim-sulfamethoxazole (66.7%), ceftazidime (33.3%), meropenem (32.05%), ertapenem (30.8%), doripenem (29.5%), imipenem, and ciprofloxacin (26.9%).

### 2.2. Multiplex PCR Detection of Carbapenem Resistance Encoding Genes (*bla*_IMP_, *bla*_NDM_, *bla*_KPC_, and *bla*_OXA_)

**Figure 1:**
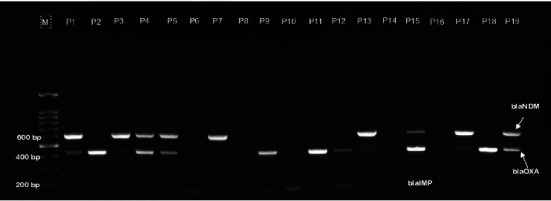
Multiplex PCR results for carbapenemase genes (*bla*_KPC_, *bla*_NDM_, *bla*_OXA_ and *bla*_IMP_) of *E. coli* isolates from chicken identified as CRE phenotypically. M, 100 bp DNA marker; lanes P1-P19, test sample (*E. coli*) isolates.

The PCR result confirmed the presence of carbapenemase genes in the identified *E. coli* isolates. Out of the 78 *E. coli* isolates, 19 (24.36%) were positive for at least one of the carbapenemase genes. Among these, about 58% (11/19) were positive for multiple carbapenemase genes. Four isolates harbored all *bla*_NDM_, *bla*_OXA_, and *bla*_IMP_, and five and two different isolates harbored *bla*_NDM_ and *bla*_OXA_ and *bla*_OXA_ and *bla*_IMP_, respectively. However, none of the isolates were positive for *bla*_KPC_ (Figure 1 and Table 1).

### 2.3. Phylogenetic Analysis

**Figure 2:**
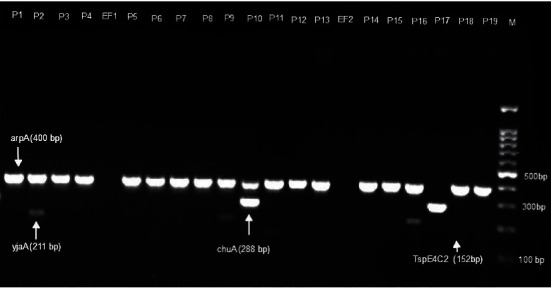
Quadruplex PCR profiles of new Clermont phylotyping method. Group A (P3, P4, P5, P6, P7, P8, + - - -); group B1 (P1, P11, P13, P18, + - - +); group C (P2, P9, P16, + - + -); group E (P10, + + - -); unknown (P17, - + - -); group D (P10, + + - -); lanes EF1 and EF2 (*Escherichia fergusonii* - - - -).

The results from quadruplex PCR showed that the CREC belong to diverse phylogroups including group A, group B1, group C, group E, group D, and group unknown. Among the 19 CREC isolates, nine were identified as members of group A while five, three, and one were, respectively, typed as group B1, group C, group D, and unknown group (Figure 2 and Table 1).

## 3. Discussion

Carbapenem resistance in common bacterial pathogens has become one of the most concerning global public health issues since the carbapenem antibiotics are among the most critically important antimicrobials for the treatment of infections in humans [9]. Carbapenems have been reported to show the broadest spectrum of antimicrobial activity *in vitro* against Gram-positive and Gram-negative bacteria, including anaerobes [10]. Because of their broad spectrum of actions, potency, and effectiveness in treating broad range of infections in humans, carbapenems have been recognized as the antibiotics of last resort to treat infections caused by multidrug-resistant Gram-negative bacteria [11]. Although carbapenemases have been known to be new and potentially emerging problem in food-producing animals, the prevalence of carbapenem resistance in bacteria from animals has been scarcely reported [9]. So far, most of the epidemiological studies and the significance of CRE have been focusing on human studies and the studies conducted in food animals have been very few. The current study reports relatively higher prevalence of CRE, 24.36% (19/78) of the total *E. coli* isolated from 150 cloacal swab samples collected from broiler chicken from commercial farms based on phenotypic identifications of which 17 were positive for the tested carbapenemase genes, whereas the two isolates were negative for carbapenemase genes while showing CREC-positive results on MIC test by using E-test strips. The meropenem, imipenem, and ertapenem MIC values for the isolates ranged from 2 *μ*g/mL to ≥256 *μ*g/mL. Most of the *E. coli* isolates were resistant to at least two antibiotics including meropenem, ertapenem, and imipenem showing multidrug resistance. The majority, about 58% (11/19) of the confirmed CREC isolates, harbored multiple carbapenemase genes. Four isolates harbored *bla*_NDM_, *bla*_OXA-48_, and *bla*_IMP_ genes, and five and two different isolates harbored *bla*_NDM_ and *bla*_OXA-48_ and *bla*_OXA-48_ and *bla*_IMP_, respectively. In general, there is scarcity of reported data on the prevalence of CREC in food animals in Malaysia. A recent study investigated the prevalence of CREC in broiler chickens, ruminants, and swine from different farms in Terengganu state. This study reported a much lower CREC prevalence of 1% (2/200) from broiler chicken cloacal swab samples collected antemortem from an abattoir in Terengganu. The same study also investigated the prevalence of CREC in 151 ruminants and 100 swine faecal samples collected from different farms in the state of Terengganu; however, no CREC was reported. A study from Egypt conducted on CRE particularly on carbapenem-resistant *K. pneumoniae* in broiler chickens from different farms, drinking water from the farms, and workers handling the chickens reported a prevalence rate of 15% and 6% from the broilers and water samples, respectively. Among the poultry CRE isolates (*n* = 15), all were *bla*_NDM_ positive, while *bla*_KPC_, *bla*_OXA48_, and *bla*_NDM_ genes were detected in 11 of the isolates while four isolates were positive for either *bla*_KPC_ or *bla*_NDM_ or *bla*_OXA-48_ and *bla*_NDM_. In addition, 56% of *K. pneumoniae* isolates from humans harboring multiple genes suggesting a high incidence of this resistant bacteria in humans may contribute to its dissemination among food-producing animals and the livestock environment, thus increasing the risk of foodborne transmission to the consumers [7]. The presence of carbapenem resistance in bacteria from animals, including food-producing animals (pigs, bovines, and horses), has also been reported from some European countries such as Germany, France, and Belgium and Egypt [12, 13]. The identification of *E. coli* isolates harboring multiple (at least two) carbapenemase-encoding genes from food animal in this study differentiates it from previous similar studies which mostly reported *E. coli* isolates harboring one or two carbapenemase genes [14, 15].

Carbapenems are not routinely used in food animal production including poultry farming; however, carbapenem resistance in the *E. coli* isolates might have coevolved along with resistance to other antibiotics that are commonly used against resistant strains of bacteria that may also be disseminated through direct contact, insect vectors, and other animals [11, 16, 17]. An earlier study by Poirel et al. [18] also suggested that coselection of carbapenemase genes under the selection pressure imposed by the use of aminopenicillins and aminopenicillin-*β*-lactamase inhibitor combinations in livestock may lead to the emergence and spread of carbapenem resistance. Reports from previous studies indicated that CRE can persist in animal production if the bacteria are adapted to animals and the farm environment and are stabilized by coexpression of further resistance genes [19, 20]. The possibility that infected or carrier humans, particularly by the farm workers, might spread resistant bacteria in farms through direct and indirect routes of transmission cannot be ruled out. This is due to the fact that humans, the farm workers in the context of the current study, are more likely to have been exposed to broad-spectrum antibiotics, and in particular to broad-spectrum *β*-lactams, than the chickens [17]. Since CREC can transmit through direct anthropozoonotic or zooanthroponotic routes [21], the spread of CREC in humans may pose risk for food animal production and possibly lead to the establishment of CREC in the food animal production ecosystem and may lead to subsequent further spread of these pathogens [19].

Phylogenetic grouping showed that the CREC isolates belonged to five different groups, groups A (47.37%), B1 (26.32%), C (15.79%), D (5.26%), and unknown (5.26%). In agreement with the current findings, a study by Asadi et al. [22] reported that the majority (54.21%) of *E. coli* isolates from chickens belonged to phylogroup A. However, contrary to the findings in this study, the authors reported that 32.53% and 7.22% of the *E. coli* isolates belonged to phylogroups D and B1, respectively. Coura et al. [23] reported that phylogroups A followed by B1 are the most common phylogroups of *E. coli* obtained from broiler carcasses suggesting the possibilities of contamination by commensal strains of *E. coli*. Cordoni et al. [24] reported that out the 272 *E. coli* strains analyzed, 132 were grouped in the B2 phylogroup, 61 in A1, 37 in group A, and 21 in groups B1 and D while the remaining 21 were not ascribable to any group. Ramadan *et al*. [25] also reported that higher frequencies of virulent phylogroups of D and B2 were found among avian pathogenic *E. coli* (APEC) isolates and phylogroup A in 25% of APEC isolates, which is predominantly associated with commensal *E. coli* which might have originated from commensal *E. coli* strains that might have acquired virulence-related genes. Interestingly, previous studies by Walk et al. [26] demonstrated that most *E. coli* strains that are able to persist in the environment belong to the B1 phylogenetic group. Earlier studies classifying the different *E. coli* phylogroups reported that the extraintestinal pathogenic strains usually belong to groups B2 and D, the commensal strains to groups A and B1, while the intestinal pathogenic strains belong to groups A, B1, and D [27]. In this study, discrepancies between the different methods for CRE detection have been observed. Some of the isolates appeared to show susceptibility towards the tested carbapenem antibiotics when tested by disc diffusion but were confirmed to be resistant as seen from the results from MIC determination by E-test and PCR detection of carbapenemase genes, whereas two isolates which showed phenotypic resistance to carbapenems did not harbor any of the carbapenemase genes tested in this study. These discrepancies can be attributed to the different levels of discriminatory abilities of the tests. In general, antimicrobial susceptibility by disc diffusion is the least reliable compared to MIC determination and PCR. Both phenotypic and molecular detection and characterization of CRE have their respective limitations and reliable monitoring of CRE from animals requires a combination of molecular and culture-based methods [21].

## 4. Materials and Methods

### 4.1. Ethics

This research was reviewed and approved by the animal research ethics committee at the Faculty of Veterinary Medicine, Universiti Malaysia Kelantan.

### 4.2. Sample Collection and Processing and Bacterial Isolation and Identification

A total of 150 samples of cloacal swabs from live chickens in poultry farms in Kelantan were collected and placed in transport media. All the samples were collected aseptically and were placed in an icebox during transportation and stored in a refrigerator at 4°C overnight and were processed the following day. The cloacal swabs were placed in 10 mL of Phosphate-buffered Saline (PBS) for enrichment and were aerobically incubated for 24 h at 37°C. The enriched samples were cultured on Nutrient agar (Oxoid, UK) and MacConkey (Oxoid, UK) agars were incubated at 37°C for 24 h. Following primary culture, bacterial growths showing lactose fermentation on the MacConkey agar (Oxoid, UK) and Gram negative were subcultured on MacConkey (Oxoid, UK) agar and Nutrient agar to obtain pure colonies. Following secondary culture, lactose-fermenting colonies on MacConkey agar were selected subcultured on Eosin Methylene Blue (EMB) (Oxoid, UK) agar 24 h at 37°C. Bacterial colonies with green metallic sheen on EMB agar were screened, and further biochemical tests were conducted to presumptively identify *E*. c*oli* isolates. Further confirmation of *E. coli* was done by PCR detection of *E. coli* species-specific gene. All the confirmed *E. coli* isolates were inoculated onto chromogenic selective agar, Brilliance™ CRE (Oxoid, UK) selective agar. Inoculated plates were incubated overnight at 37°C, and presumptive CRE *E. coli* were identified according to the manufacturer's guideline. Colonies with blue or pale pink colours were presumptively identified as CRE. All the isolates which did not show the expected colonial morphologies of CRE were further tested by PCR amplifications of common carbapenemase-encoding genes.

### 4.3. Antibiotic Sensitivity Test (AST)

Antibiotic sensitivity test was done using Kirby-Bauer disk diffusion method on Mueller-Hinton Agar (MHA) (Oxoid, UK) with all the identified isolates according to the Clinical and Laboratory Standards Institute (CLSI) guidelines [28]. *Escherichia coli* ATCC 25922 strain was used as quality control. Disc diffusion method was used to determine antimicrobial susceptibility test, and the antibiotic discs used were streptomycin (10 *μ*g), gentamycin (10 *μ*g), enrofloxacin (5 *μ*g), ciprofloxacin (5 *μ*g), trimethoprim sulfamethoxazole (25 *μ*g), ceftazidime (30 *μ*g), ceftriaxone (30 *μ*g), imipenem (10 *μ*g), meropenem (10 *μ*g), ertapenem (10 *μ*g), and doripenem (10 *μ*g). The media were incubated for 24 h at 37°C. After incubation, zone of inhibition for each of the antibiotic discs was measured and the antibiotic susceptibility was determined based on CLSI guidelines [28].

### 4.4. Determination of Minimum Inhibitory Concentration (MIC) Using E-Test

The MIC determination using E-test (Biomerieux, France) was done as recommended by the manufacturer. Briefly, overnight culture of *E.coli* was suspended in 10 mL normal saline (0.9% NaCl). The turbidity of the bacterial suspension was adjusted to that of 0.5% McFarland standard. The bacterial suspension was then uniformly streaked onto the entire surface of MHA (Oxoid, UK). Interpretations of the E-test strips (Biomerieux, France) were done according to the CLSI standards [28]. *Escherichia coli* ATCC 25922 strain was used as quality control.

### 4.5. Molecular Characterization of Carbapenem-Resistant E. coli

#### 4.5.1. DNA Extraction

Following bacterial isolation and identification, genomic DNA extraction was performed for all the presumptive *E. coli* isolates using boiling method. One to two bacterial colonies from each of the isolates on Nutrient agar were resuspended into a 1.5 mL microcentrifuge tube containing 100 *μ*L of 10 mmol/L Tris-HCl buffer (pH 8.0). The microcentrifuge tubes containing the samples were vortexed, and the suspensions were then boiled for 10 minutes to lyse the cells, followed by quickly chilling on ice for 5 minutes. Then, the tubes containing the suspensions were centrifuged at 12000 rpm for 10 minutes. Following that, 100 *μ*L of the supernatant containing DNA from each of the microcentrifuge tubes was transferred into another 1.5 mL microcentrifuge and the DNA quality was assessed using a spectrophotometer and stored at -20°C until further use.

#### 4.5.2. Molecular Detection of E. coli and Carbapenem Resistance Encoding Genes

PCR amplification was conducted to identify *E. coli* using primer Pho-F/Pho-R targeting the housekeeping genes of *E. coli* and carbapenemase-encoding genes (*bla*_NDM_, *bla*_OXA_, *bla*_IMP_, and *bla*_KPC_) as described earlier [1, 29]. The PCR reaction mixture was prepared in a 0.5 mL Eppendorf tube prior to addition of templates. Each microcentrifuge tube contained 25 *μ*L of the PCR Master Mix, 1 *μ*L of 10 *μ*m each primer, and 18 *μ*L of sterile nuclease-free water. Then, 5 *μ*L of DNA template was added to each tube. Sterile nuclease-free water was used as negative control. All PCR amplifications were conducted using Thermal Cycler 1000 (Bio-Rad, USA). Amplification of an *E.coli*-specific gene (*pho*) was carried out using the following protocol: initial denaturation for 2 mins at 94°C, followed by 35 cycles consisting final denaturation for 1 min at 94°C, primer annealing for 1 min at 56°C, DNA extension for 1 min at 72°C, followed by final extension for 10 mins at 72°C, and holding at 12°C.

For the amplification of carbapenem-resistant genes, the PCR reaction constituted 25 *μ*L of the 2x PCR Master Mix (Promega, USA), 1 *μ*L of 10 *μ*m of the three primer pairs, and 14 *μ*L of sterile nuclease-free water. Amplification of carbapenem-resistant genes was conducted by the following thermal cyclic conditions: activation of thermostable hot-start DNA polymerase for 10 mins at 94°C, followed by 36 cycles of amplification consisting of denaturation for 30 s at 94°C, primer annealing for 40 s at 52°C, and strand elongation for 50 s at 72°C, with 5 mins at 72°C for the final extension, and holding for 12°C. Analysis of the PCR amplification products was done by using electrophoresis in a 1.5% agarose gel at 100 V and 400A for 40 mins in 1× TBE buffer. The DNA fragments were then visualized using GelDoc EZ Imager (Bio-Rad, USA). The DNA size was determined using the 100 bp molecular weight ladder as a marker.

#### 4.5.3. Phylogenetic Analysis

Characterization of the phylogenetic groups of the *E. coli* isolates was determined according to the protocols described by Clermont et al. [30]. Briefly, a single PCR reaction mixture contains 12.5 *μ*L of 2x DreamTaq Master Mix (Promega, USA), 5 *μ*L of DNA (approximately 100 ng), and 20 *μ*M of each primer in a total volume of 30 *μ*L. PCR amplifications were carried out in a Nexus Gradient Mastercycler (Eppendorf, USA) using the following conditions: initial denaturation at 94°C for 4 min and 30 cycles for each denaturation at 94°C for 5 s annealing at 57°C for 20 s (group E) or 59°C for 20 s (quadruplex and group C), amplification at 72°C for 1 min, and final extension at 72°C for 5 min. The PCR products were analyzed by electrophoresis in 1.5% agarose gel, and image analysis was done using GelDoc™ EZ Imager (Bio-Rad, USA).

## 5. Conclusions

In conclusion, the detection of CREC in this study shows that these resistant bacteria are not limited to human infections and that CREC has also become an emerging problem in farm animals, particularly in chicken farms. This may raise concerns that these carrier food animals may serve as a source of infection and/or colonization for humans. This implies the potential public health risks posed by emerging antimicrobial resistance particularly CREC in food animals and the need for appropriate control and prevention measures to minimize the spread of such resistant bacteria.

## Figures and Tables

**Table 1:** Antimicrobial resistance profile and phylogenetic diversity of CRE isolated from cloacal swab samples from chicken.

Isolate ID	Antimicrobial susceptibility (disc diffusion)			E-test MIC value			Carbapenem resistance (carbapenemase-encoding genes)				Phylogroup
	ETP (10 *μ*g)	MEM (10 *μ*g)	IMP (10 *μ*g)	ETP (*μ*g/mL)	MEM (*μ*g/mL)	IMP (*μ*g/mL)	*bla*_KPC_	*bla*_NDM_	*bla*_OXA_	*bla*_IMP_	
P1	S	R	R	8	≥256	4	-	+	+	-	B1
P2	S	S	R	6	8	16	-	-	+	-	C
P3	R	R	R	32	≥256	32	-	+	+	+	A
P4	R	R	R	4	≥256	4	-	+	+	-	A
P5	R	S	R	8	32	32	-	+	-	-	A
P6	R	R	R	4	6	8	-	-	+	-	A
P7	R	R	R	2	32	32	-	+	+	+	A
P8	S	S	R	4	6	32	-	-	-	-	A
P9	R	S	S	32	8	32	-	-	+	+	C
P10	R	R	R	16	32	≥256	-	+	+	+	D
P11	R	R	R	4	0.25	32	-	-	-	+	B1
P12	R	S	R	8	16	8	-	-	+	+	A
P13	R	R	R	2	4	8	-	+	+	+	B1
P14	R	R	R	2	1.5	6	-	-	-	+	A
P15	S	R	R	0.25	≥256	4	-	+	+	-	B1
P16	S	S	R	6	4	8	-	-	-	-	C
P17	R	S	R	0.25	32	6	-	+	+	-	Unknown
P18	S	S	R	2	16	0.25	-	-	+	-	B1
P19	S	S	R	0.25	≥256	≥256	-	+	+	-	A

## Data Availability

All the relevant data have been included in the manuscript.
